# A case report of adult juvenile polyposis syndrome with SMAD4 pathogenic variant

**DOI:** 10.3389/fonc.2023.1114097

**Published:** 2023-03-06

**Authors:** Yutong Liu, Zeyu Wang, Zhongyu Zhang, Yuanyuan Sun, Yanyan Zhang, Jiamei Yang

**Affiliations:** Department of Oncology, The Second Affiliated Hospital of Zhengzhou University, Zhengzhou, China

**Keywords:** gastric polyposis, juvenile polyposis, juvenile polyposis syndrome, SMAD4 pathogenic variants, case report

## Abstract

**Background:**

Juvenile polyposis syndrome (JPS) is a rare autosomal dominant disorder that is a type of hamartomatous polyp syndrome, and its incidence rate is approximately 1/100000. The main clinical feature is the presence of multiple juvenile polyps in the gastrointestinal tract, most often in the colorectal tract. We present a case of juvenile polyposis syndrome with massive gastric polyposis.

**Case presentation:**

A 50-year-old male was admitted to the hospital due to abdominal distension and poor appetite. Gastroscopy revealed a large number of gastric polyps. Pathological findings revealed gastric juvenile polyps. Genetic testing revealed that he and his brother both carried *SMAD4*: c.266_269del germline pathogenic variant. The final diagnosis was juvenile polyposis syndrome of the stomach. He once suffered from colon cancer and bladder cancer. One of his brothers died of colon cancer, and the other brother suffered from colon polyps.

**Conclusions:**

Gastric involvement in juvenile polyposis syndrome is relatively rare. When massive gastric polyposis is found, gene detection should be carried out as soon as possible, so that rapid diagnosis and treatment can be obtained.

## 1 Introduction

Juvenile polyposis syndrome (JPS) is a rare autosomal dominant disorder that is a type of hamartomatous polyp syndrome, and its incidence rate is approximately 1/100000 (1). The main clinical feature is the presence of multiple juvenile polyps in the gastrointestinal tract, most often in the colorectal (98%) but also in the stomach (14%) and small intestine (9%) (2, 3).

Pathogenic variant in the *SMAD* family member 4 (*SMAD4*) gene on chromosome 18 or the bone morphogenetic protein receptor 1A (*BMPR1A*) gene on chromosome 10 are detected in approximately 50%-60% of patients clinically diagnosed with JPS (4–6). *BMPR1A* is a serine threonine kinase type I receptor. The activated receptor phosphorylates and activates the downstream cytoplasmic *SMAD* protein, participating in the transforming growth factor-β (TGF-β) superfamily signaling pathway, thereby affecting cell growth, differentiation, apoptosis and other processes (7–9).

Herein, we introduce a JPS patient with massive gastric polyposis carrying *SMAD4* gene pathogenic variant.

## 2 Case presentation

**Figure 1:**
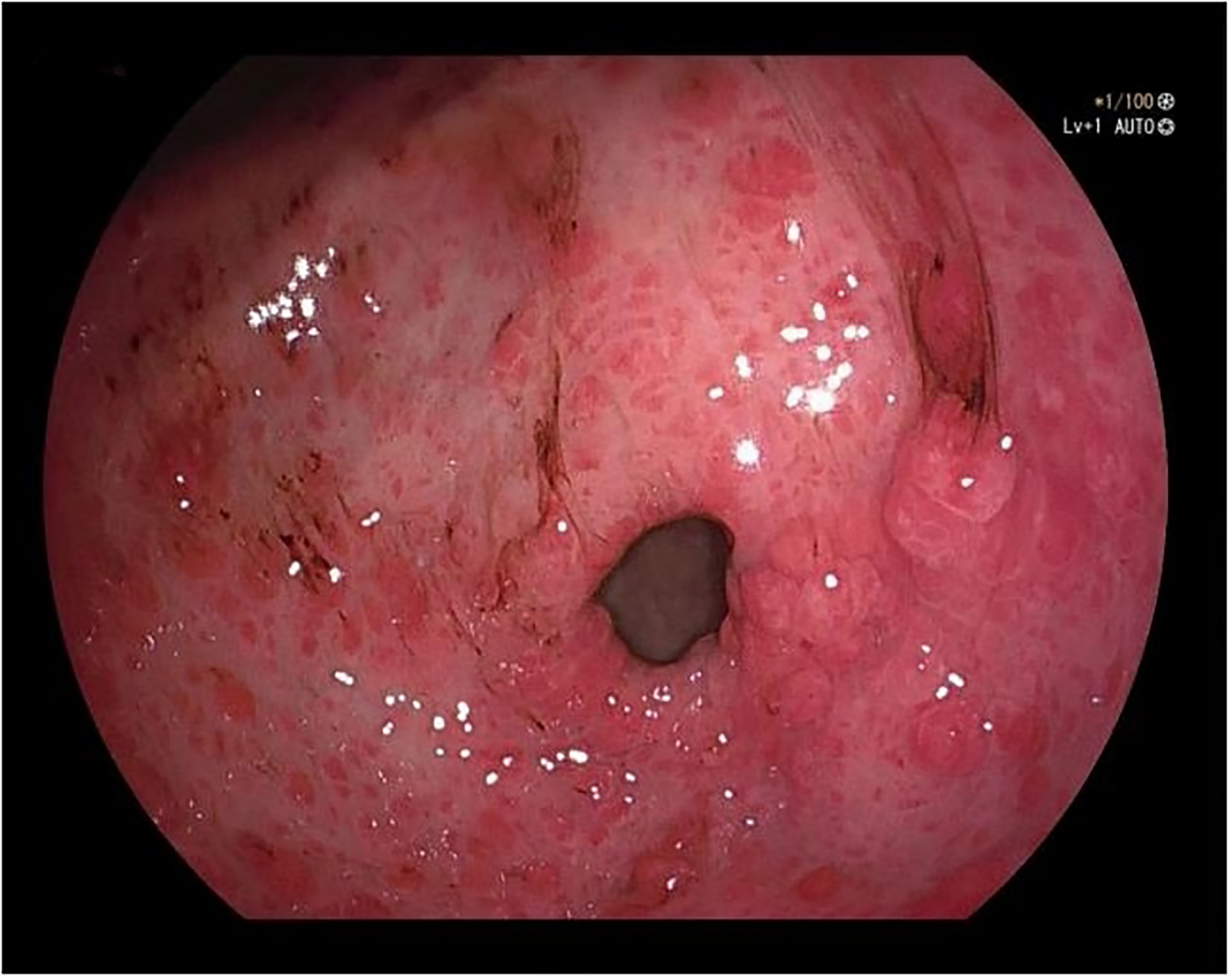
Gastric antrum.

**Figure 2:**
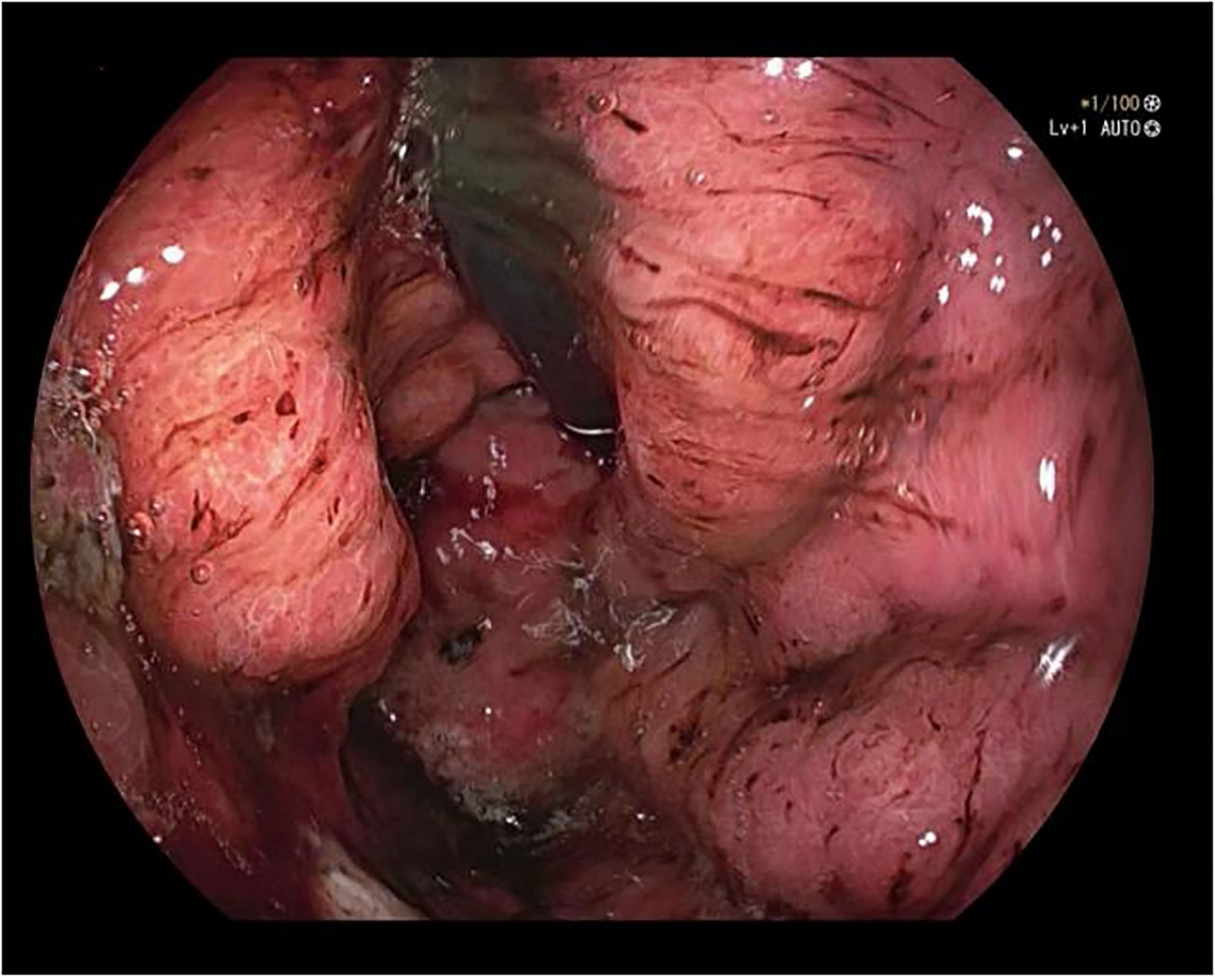
Gastric fundus.

**Figure 3:**
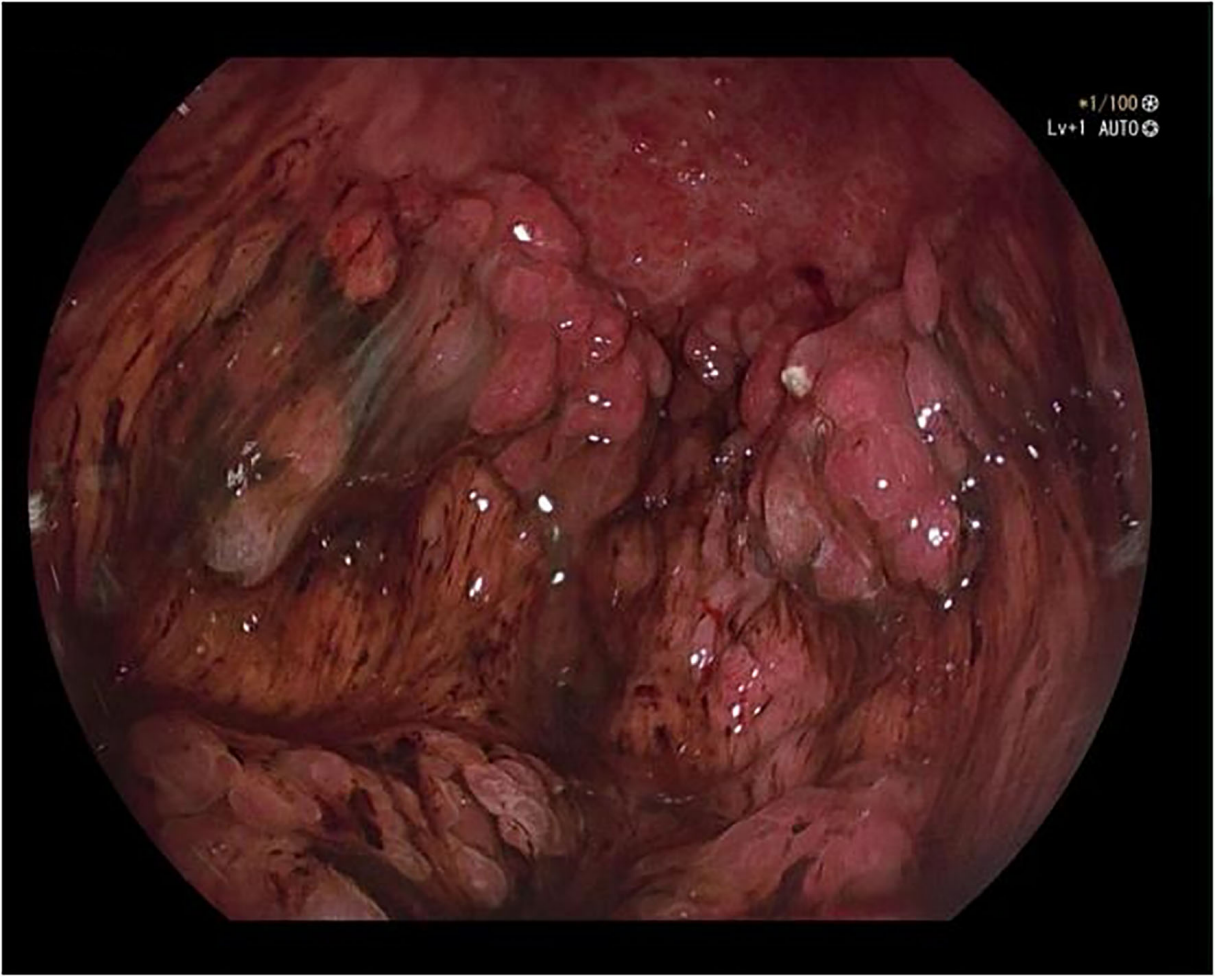
Gastric body.

**Figure 4:**
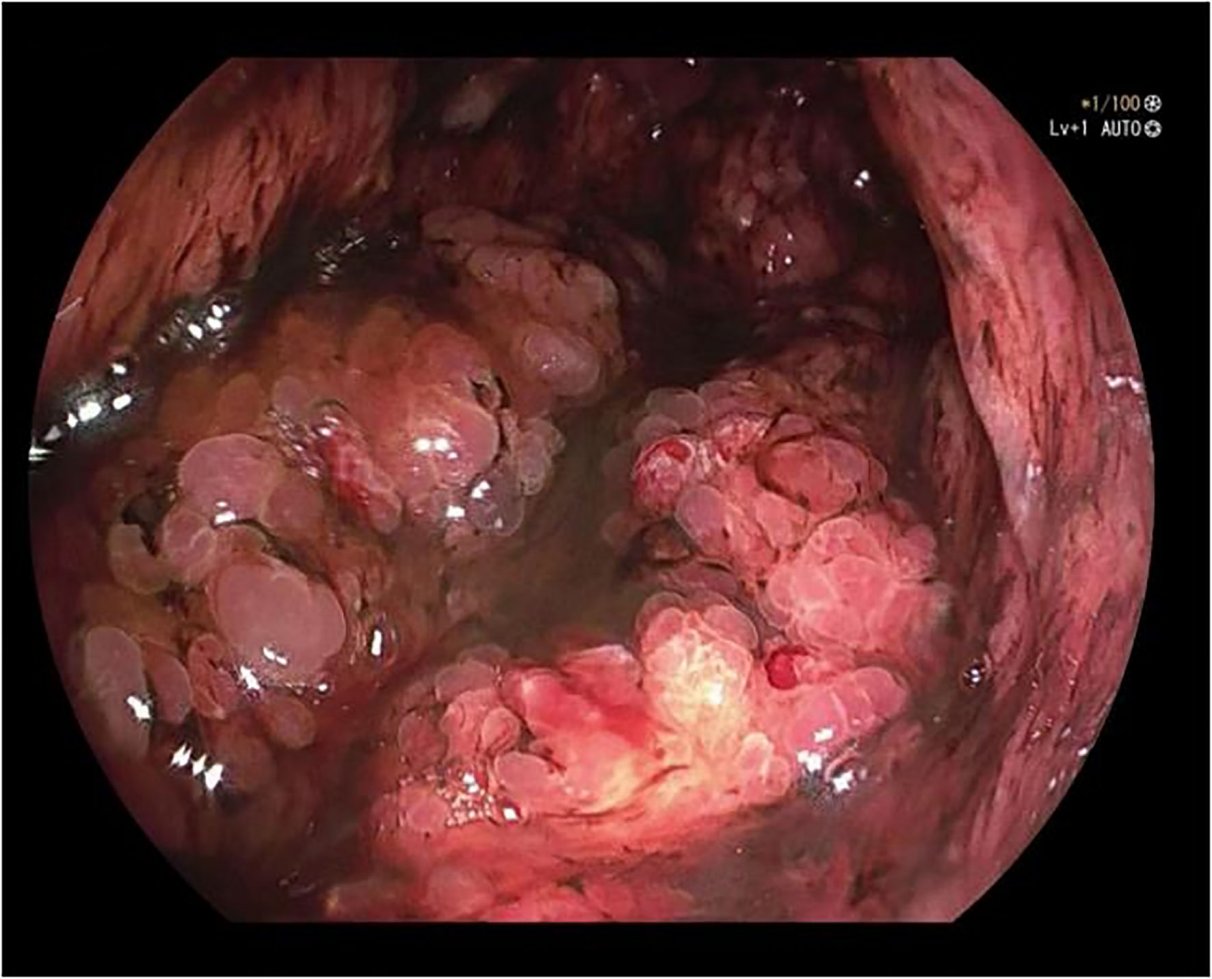
Gastric body.

**Figure 5:**
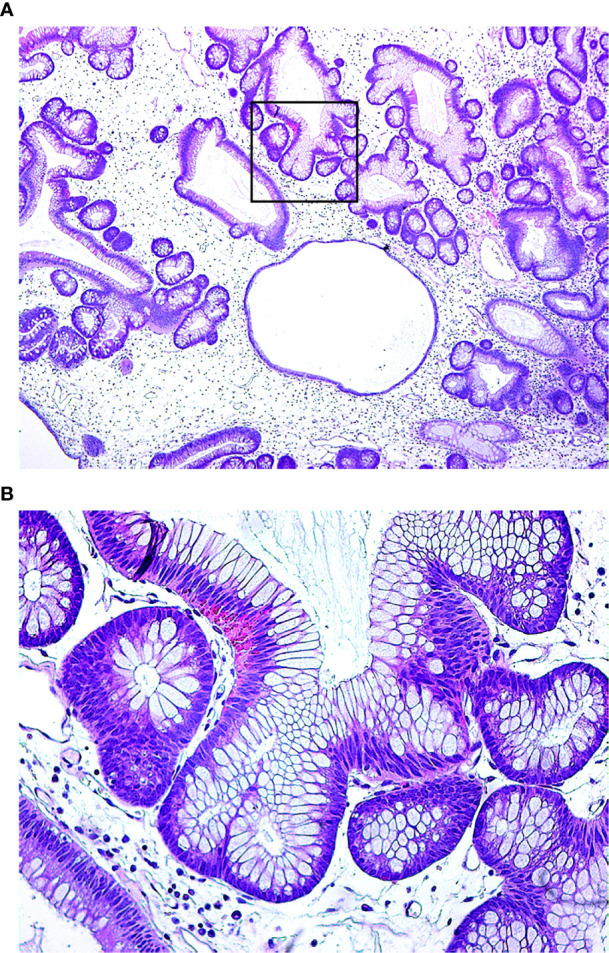
Under the microscope, the glandular cavity of the gastric body is irregular in shape, manifested as expansion or distortion, and there are small glandular cavities around, mucosal interstitial edema, accompanied by infiltration of chronic inflammatory cells. (**A**. 40×; **B**. 200×).

A 50-year-old male was admitted to the hospital due to abdominal distension and poor appetite. Laboratory examination results show that hemoglobin of 62 g/L, albumin of 32.2 g/L and the stool routine occult blood test was weakly positive. In order to further clarify the cause, the patient was examined by gastrointestinal endoscopy. Gastrointestinal endoscopy showed that the whole gastric mucosa was nodular hyperplasia, protuberance, densely distributed, and clustered locally, mainly in the fundus of the stomach. Massive hyperplasia lesions were removed at the endoscopic mucosal resection (EMR) of gastric body lesions for examination. A mass of nodular hyperplasia was found in the fundus of the stomach, and the structure of the top gland was lost. Two biopsies were taken. A large diverticulum can be seen in the descending colon, with smooth mucosa, and no bleeding, erosion, ulcer or other lesions are found. A pedunculated polyp of 1.0*2.0 cm in size can be seen in the sigmoid colon. The surface mucosa was congestive and edematous with erosion. A snare device was used to snare, and high-frequency electric resection was performed. The other colonic and rectal mucosa were smooth, with clear vascular texture and no obvious abnormalities. **Figures 1**–**4** show the patient’s condition under a gastroscope. Pathological findings suggest the following: 1. (gastric fundus biopsy) mucosa high-grade intraepithelial neoplasia; 2. (gastric biopsy) juvenile polyps (**Figure 5**); 3. (sigmoid colon biopsy) Low-grade tubular adenoma with clean margins. Immunohistochemical results showed CK8/18 (+), Ki-67 (+50%), P53 (-, mutant), and HP (–).

**Figure 6:**
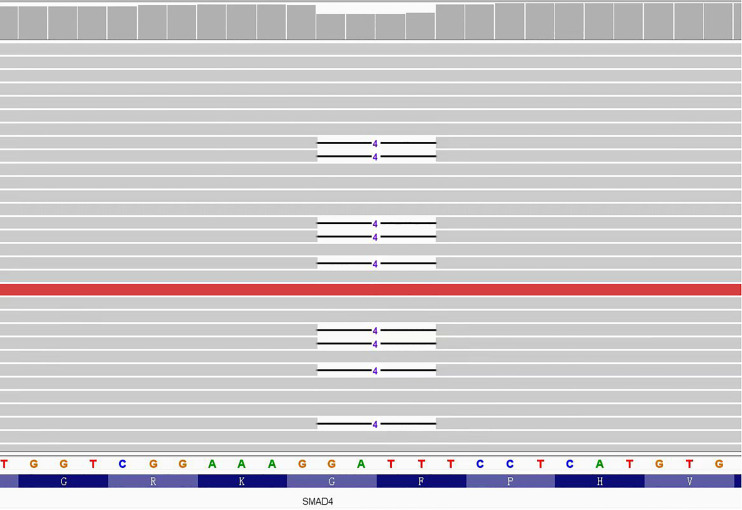
Schematic diagram of *SMAD4* gene pathogenic variant.

According to the pathological results, JPS was suspected, so we suggested that the patient and his brother should carry out gene testing to determine whether there is gene mutation. Gene testing of the patient and his brother showed that the *SMAD4*: c.266_269del pathogenic variant was present in exon 3 of the *SMAD4* gene (**Figure 6**). It is a frameshift mutation caused by the deletion of base 266-269, resulting in the change of glycine at position 89 to valine (*SMAD4*: p.G89Vfs*4). This pathogenic variant will lead to early termination of translation, encoding truncated protein products and inactivating *SMAD* protein complexes, thus affecting the TGF-β signaling pathway.

In combination with clinical pathology and genetic testing, the patient was diagnosed with gastric juvenile polyposis syndrome with *SMAD4* gene pathogenic variant. It is recommended that the patient undergo preventive total gastrectomy. Because cystoscopy indicates bladder tumor recurrence, bladder tumor resection should be performed first. The patient was advised to follow up with gastroscopy and to undergo surgery in a timely manner.

Past history: The patient had undergone “partial cystectomy+cystectomy+left ureteroplasty+left ureteral stenting tube placement” due to “uroepithelial papillary tumor”, and “laparoscopic right hemicolectomy” due to “well-differentiated adenocarcinoma of the right colon”. Family history: One brother of the patient died of colon cancer and the other brother suffered from colon polyps.

## 3 Discussion

The clinical diagnosis of JPS was mainly based on the Jass diagnostic criteria: ① The number of juvenile colorectal polyps ≥5; ② Multiple juvenile polyps in the whole gastrointestinal tract; ③ Any number of juvenile polyps with a family history of juvenile polyps, which can be diagnosed if one of the above three manifestations is met (10). However, in this case, the patient did not fully meet the diagnostic criteria, and the final diagnosis was only confirmed by genetic testing. Soer et al. reported 2 cases of multiple gastric juvenile polyps with *SMAD4* gene pathogenic variant. According to the pathological results, Menetrier disease (MD) was considered to be diagnosed at the early stage, and JPS was finally diagnosed by gene testing (11). The pathological characteristics of juvenile polyps are similar to those of proliferative polyps or inflammatory polyps, and it is difficult to distinguish them only according to their histological characteristics. Therefore, gene detection is relatively important.

At present, pathogenic variant of *SMAD4* and *BMPR1A* genes have been identified in JPS. Compared with patients with *BMPR1A* gene pathogenic variant, patients with *SMAD4* gene pathogenic variant have a higher risk of gastric polyposis and gastric cancer (5). Friedl et al. reported 29 patients with clinical diagnosis of JPS. Massive gastric polyposis was found in 7 patients with *SMAD4* pathogenic variant, and many of the families of 2 patients also suffered from massive gastric polyposis, so they underwent partial or total gastrectomy. However, no severe form of gastric polyposis was observed in patients with *BMPR1A* pathogenic variant or without mutation (12). There is also a high risk of hereditary hemorrhagic telangiectasia (HHT), which consists mainly of telangiectasia of the skin and mucosa and arteriovenous malformations of the liver, lungs and brain (13). In a retrospective study evaluating 34 patients with pathogenic variant in *SMAD4*, colon polyps were found in 31 (97%) of the 32 patients who underwent colonoscopy and gastric polyps in 21 (68%) of the 31 patients who underwent gastroscopy, while clinical features of HHT were present in 76% of patients (14). In addition, it is controversial whether pathogenic variant in the *PTEN* and *ENG* genes are associated with the development of JPS. Some investigators have suggested that JPS patients with *PTEN* pathogenic variant are only patients with Cowden syndrome (CS) or Bannayan-Riley-Rruvalcaba syndrome (BRRS), whose extraintestinal symptoms have not yet appeared (15).

Compared to the general population, the JPS has an increased risk of developing cancers such as colorectal cancer (16). Studies have shown that the incidence of colorectal and gastric cancer in JPS patients is 39% (17) and 21% (18), respectively. In addition, pancreatic, duodenal and small bowel cancers also occur in a smaller proportion of patients (19, 20). The updated data of a large retrospective study in Europe show that the incidence rate of gastric cancer of *SMAD4* pathogenic variant carriers is earlier, with a median age of 44 years (21). The mechanism of JPS carcinogenesis is still unclear. At present, some researchers have proposed that it also follows the classic model of “atypical hyperplasia - adenoma - cancer”. It is worth noting that this is the first case of a patient with JPS who has both colon and bladder cancer, but whether bladder cancer is associated with JPS remains to be investigated.

At present, there is no consensus on the treatment of JPS, which mainly depends on expert experience. Patients with JPS with few polyps can be treated with regular endoscopic polypectomy. Juvenile colorectal polyposis that cannot be controlled by endoscopic polypectomy may be considered for total or subtotal colectomy (2).Multiple diffuse gastric polyps or symptomatic familial juvenile polyposis usually also require surgical intervention, with major gastrectomy or total gastrectomy recommended (2, 22). Prophylactic total gastrectomy may be considered for patients with multiple giant gastric polyposis, as some patients will still require residual gastrectomy after subtotal gastrectomy (23).

Patients with JPS need regular follow-up and endoscopic monitoring. For patients with suspected JPS or a family history of JPS, it is recommended that gastrointestinal endoscopy be performed from the age of 12 or when symptoms first appear, and then review gastrointestinal endoscopy every 1-3 years according to the condition of polyps (2). If JPS is diagnosed, capsule endoscopy is recommended to confirm the condition of the small intestine. A retrospective multicenter study in Europe showed that the incidence of intestinal polyps in *SMAD4* carriers and *BMPR1A* carriers was 15.7% (20/127) and 3.2% (3/94), respectively (21). Screening for symptoms associated with HTT, such as chest radiography, cranial MRI and liver ultrasound for arteriovenous malformations, is recommended from 6 months of age for JPS patients with *SMAD4* pathogenic variant (24).

Regrettably, in this case, due to economic and personal reasons, the patient did not perform capsule endoscopy and gene testing of the three generations members of the family, and because of the recurrence of bladder cancer, gastrectomy was not performed, so the complete data could not be obtained.

## 4 Conclusion

Given that it is difficult to distinguish JPS with massive gastric polyposis from other gastric hypertrophic diseases, it is highly recommended to carry out gene detection at the early stage of diagnosis. At the same time, different types of gene mutations are of great significance for disease screening and treatment.

## Data availability statement

The original contributions presented in the study are included in the article/supplementary material. Further inquiries can be directed to the corresponding author.

## Ethics statement

Written informed consent was obtained from the participant/patient(s) for the publication of this case report.

## Author contributions

JY conceived of the idea and provided guidance. YL and ZW provided the first draft of the manuscript. ZZ, YS and YZ carefully reviewed and revised the manuscript. All authors contributed to the article and approved the submitted version.
